# A critical review of the progress and application of finite element models in corneal biomechanics

**DOI:** 10.3389/fbioe.2026.1850437

**Published:** 2026-10-28

**Authors:** Shuyu Zhang, Yunfei Lan, Bo Wang, Weiqing Cheng, Yongtao Lyu, Lijun Zhang, Lin Jin

**Affiliations:** 1 Department of Ophthalmology, The Third People's Hospital of Dalian, Dalian Third People's Hospital Affiliated to Dalian University of Technology, Dalian, China; 2 School of Mechanics and Aerospace Engineering, Dalian University of Technology, Dalian, China; 3 DUT-BSU Joint Institute, Dalian University of Technology, Dalian, China; 4 Liaoning Provincial Key Laboratory of Cornea and Ocular Surface Diseases, Dalian Third People’s Hospital Affiliated to Dalian University of Technology, Dalian, China; 5 Liaoning Provincial Optometry Technology Engineering Research Center, Dalian Third People's Hospital Affiliated to Dalian University of Technology, Dalian, China

**Keywords:** corneal biomechanics, corneal cross-linking, finite element analysis, keratoconus, refractive surgery

## Abstract

Corneal morphology and biomechanics are the primary determinants of visual quality. Accurate biomechanical characterization is essential for optimizing the outcomes of refractive surgery and managing pathologies such as keratoconus. Finite element modeling is an established method used to simulate surgical procedures, predict mechanical responses, and investigate pathological mechanisms. This review evaluates the progression of corneal finite element models. Early work relied on idealized two-dimensional cross-sections or simplified three-dimensional shapes; today, researchers routinely build personalized, high-resolution three-dimensional reconstructions directly from clinical image data. Material descriptions have moved from basic isotropic linear elasticity to advanced hyperelastic, anisotropic, and viscoelastic formulations that more accurately simulate the physiological corneal behavior. This review also covers concrete applications in refractive surgery planning, corneal cross-linking optimization, and mechanistic studies of corneal pathology, as well as extensions to transplantation and orthokeratology. Taken together, these models provide reliable biomechanical evidence that supports individualized surgical design, refined cross-linking parameters, and improved graft assessment, steps that can increase the predictability and safety of modern ophthalmic procedures.

## 1 Introduction

The cornea serves as the primary refractive interface of the human eye, whose biomechanical stability directly determines visual quality and surgical safety. Composed of five ultrastructural layers, the corneal stroma dominates tissue mechanics via orthogonally arranged collagen lamellae, yielding strong anisotropy and nonlinear mechanical behaviors (Figure 1). While anatomical features provide a structural basis, the primary challenge in modern corneal care lies in the inability to quantitatively predict biomechanical deterioration underlying high-incidence clinical challenges: postoperative ectasia (0.04%–0.6%) after refractive surgery, progressive protrusion in keratoconus (1:2000 prevalence), unstable graft-host integration after transplantation, unpredictable remodeling in orthokeratology, and biased intraocular pressure (IOP) measurements caused by individual stiffness variations (Regnault et al., 2023; Kory et al., 2024; Qin et al., 2025). Conventional clinical evaluations rely on morphological indices alone and cannot capture microscale collagen disruption or stress redistribution; this creates an urgent unmet need for reliable computational methods to elucidate biomechanical mechanisms and support personalized treatment.

**FIGURE 1:**
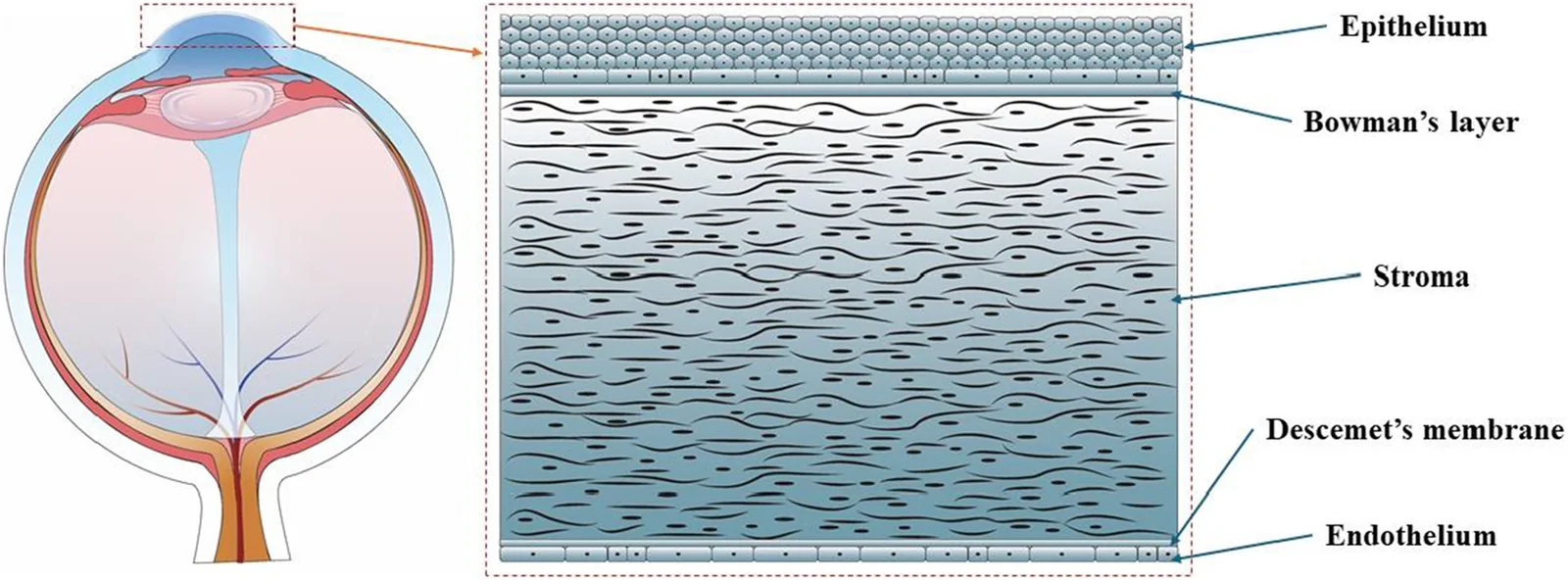
Corneal structure diagram created by the authors based on the anatomical information described in Ramasubramanian et al. (2022) and Bahramizadeh-Sajadi et al. (2024).

Finite element (FE) modeling has evolved into a gold-standard framework for *in silico* corneal biomechanics, advancing from simplified linear homogeneous representations to patient-specific, anisotropic, and multiphysics-coupled digital twins (Ji et al., 2023; De La Hoz et al., 2024; He et al., 2024; Kory et al., 2024; Zhang et al., 2025). This review systematically maps the two-decade progression of corneal FE models, covering full modeling workflows (geometry reconstruction, meshing, boundary-loading conditions, constitutive laws, and multiphysics coupling) and representative clinical applications (refractive surgery, keratoconus, cross-linking, transplantation, and orthokeratology). By synthesizing validated methods, quantitative benchmarks, and unresolved limitations, this work aims to bridge engineering simulation and clinical translation, guiding safer and more precise ophthalmic decision-making.

### 1.1 Literature search strategy

To ensure comprehensiveness and representativeness, a systematic literature search was performed across two major academic databases: Scopus and Web of Science (WOS), covering the publication window from 1 January 2007 to 4 May 2026.

Search Strings:

- Scopus:

(Cornea* OR “Keratoconus” OR “Refractive Surgery”) AND (“Finite Element” OR “FEM” OR “FEA” OR “Numerical Simulation”) AND (“Biomechanics” OR “Constitutive Model” OR “Hyperelasticity” OR “Anisotropy”) AND PUBYEAR >2006 AND PUBYEAR <2027 AND (LIMIT-TO (SRCTYPE, “j”)) AND (LIMIT-TO (DOCTYPE, “ar”) OR LIMIT-TO (DOCTYPE, “re”)) AND (EXCLUDE (SUBJAREA, “ECON”) OR EXCLUDE (SUBJAREA, “BUSI”) OR EXCLUDE (SUBJAREA, “NEUR”) OR EXCLUDE (SUBJAREA, “CENG”) OR EXCLUDE (SUBJAREA, “PHAR”) OR EXCLUDE (SUBJAREA, “AGRI”) OR EXCLUDE (SUBJAREA, “ENVI”) OR EXCLUDE (SUBJAREA, “ENER”) OR EXCLUDE (SUBJAREA, “ARTS”) OR EXCLUDE (SUBJAREA, “NURS”) OR EXCLUDE (SUBJAREA, “DENT”) OR EXCLUDE (SUBJAREA, “PSYC”) OR EXCLUDE (SUBJAREA, “SOCI”) OR EXCLUDE (SUBJAREA, “BIOC”) OR EXCLUDE (SUBJAREA, “EART”) OR EXCLUDE (SUBJAREA, “VETE”) OR EXCLUDE (SUBJAREA, “IMMU”)) AND (LIMIT-TO (LANGUAGE, “English”)) AND (LIMIT-TO (EXACTKEYWORD, “Biomechanics”) OR LIMIT-TO (EXACTKEYWORD, “Finite Element Analysis”) OR LIMIT-TO (EXACTKEYWORD, “Cornea”) OR LIMIT-TO (EXACTKEYWORD, “Keratoconus”) OR LIMIT-TO (EXACTKEYWORD,“Intra Ocular Pressure”) OR LIMIT-TO (EXACTKEYWORD, “Collagen”) OR LIMIT-TO (EXACTKEYWORD, “Finite Element Method”) OR LIMIT-TO (EXACTKEYWORD, “Elasticity”) OR EXCLUDE (EXACTKEYWORD, “Glaucoma”)).

- Web of Science (WOS):

(Cornea* OR “Keratoconus” OR “Refractive Surgery”) and (“Finite Element” OR “FEM” OR “FEA” OR “Numerical Simulation”) and (“Biomechanics” OR “Constitutive Model” OR “Hyperelasticity” OR “Anisotropy”).

Refined constraints: Publication year 2007–2026; Document types: Article or Review Article; Language: English.

Using the above search strategy, 955 documents were retrieved from Scopus and 184 from WOS. After removing duplicates, manual screening was performed (Figure 2), resulting in 243 documents for final inclusion. During the reading of these documents, it was found that within the time span from 2021 to 2025, a large number of documents focused on refractive surgery (25 articles): photorefractive keratectomy (PRK), laser-assisted *in situ* keratomileusis (LASIK) and small incision lenticule extraction (SMILE), keratoconus (KC)/corneal cross-linking (CXL) (39 articles), transplantation (3 articles): penetrating keratoplasty (PK), deep anterior lamellar keratoplasty (DALK), descemet stripping endothelial keratoplasty (DSEK), and orthokeratology (OK Lens) (7 articles).

**FIGURE 2:**
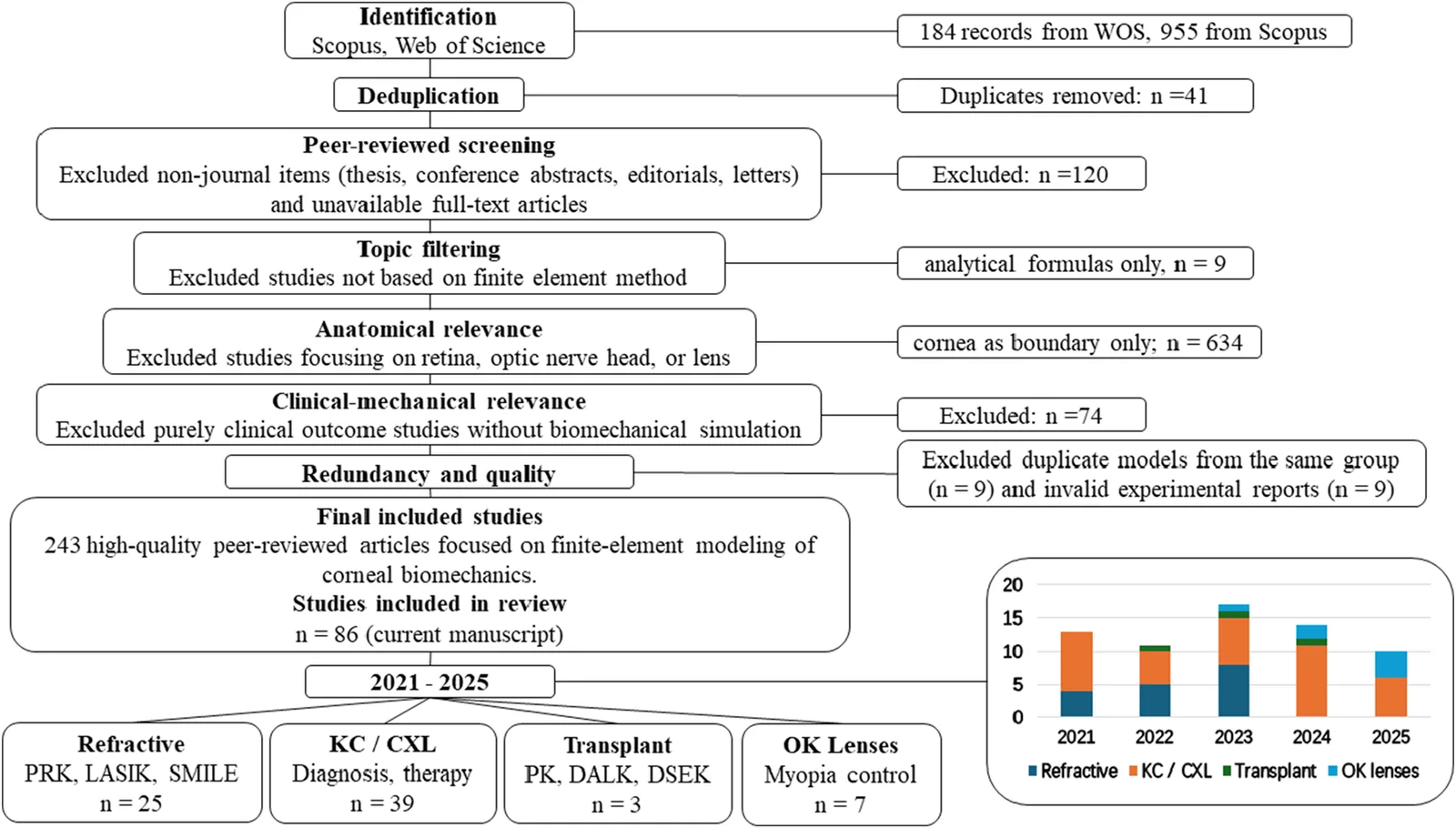
Diagram for the literature search flow.

Among the 243 eligible studies, 86 high-quality papers were selected for citation and detailed discussion based on strict screening criteria: (1) methodological innovation and completeness of finite element model validation, (2) high clinical relevance to corneal biomechanics applications, (3) publication recency (focusing on 2021–2025, the period of rapid development), (4) representativeness of modeling workflows and clinical scenarios, and (5) exclusion of duplicate or low-methodological-quality studies from the same research group. This selection ensures the review focuses on core progress, key methodologies, and typical clinical applications without redundancy.

## 2 Corneal finite element modeling workflow

Building a high-fidelity corneal model follows a consistent logic: clinical imaging drives geometry, which is then turned into a complete mechanical simulation. The five main steps: geometry, meshing, boundary/loading conditions, constitutive model, and multi-physics coupling-form the foundation for the applications discussed later (Figure 3).

**FIGURE 3:**
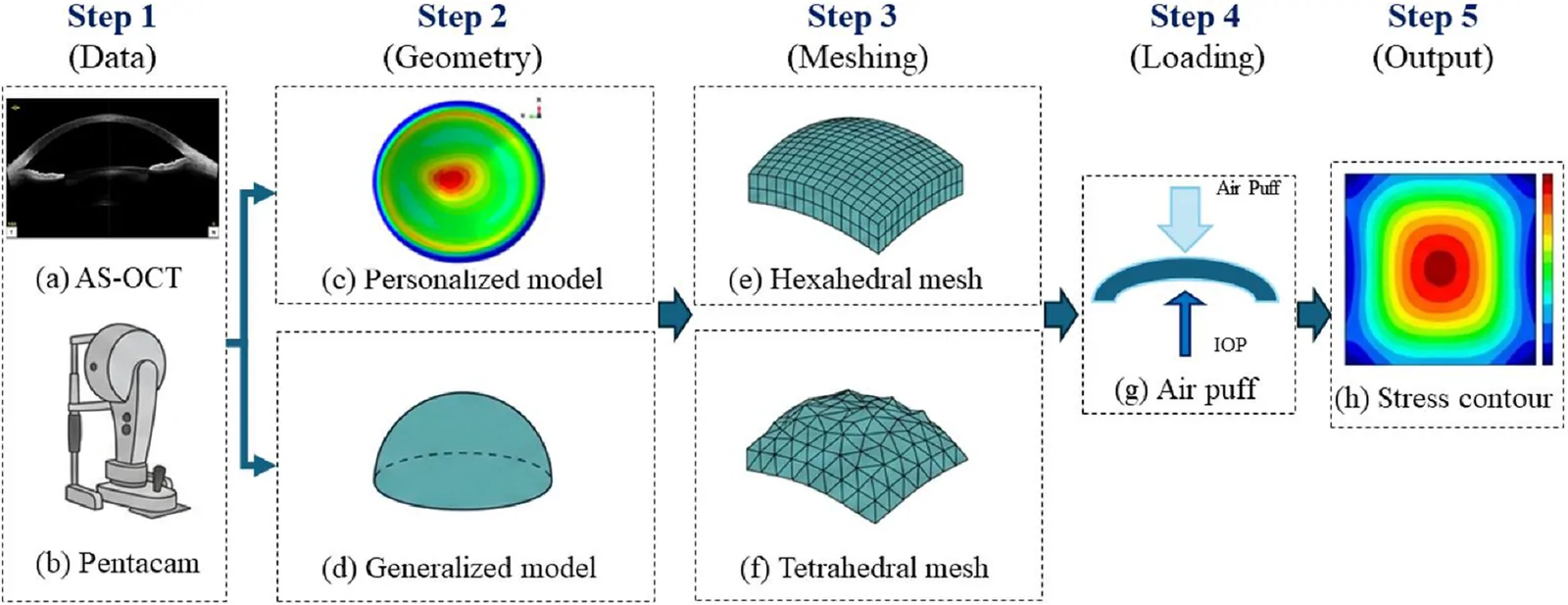
Corneal finite element modeling workflow: **(a)** Anterior segment optical coherence tomography (AS-OCT), original image provided by co-author Lin Jin; **(b)** Pentacam, author-created schematic based on the scientific information reported in Doll et al. (2020); **(c)** personalized model, adapted from Bagheri et al. (2021) under a Creative Commons Attribution (CC BY) license, with cropping and resizing only; **(d)** generalized model, author-created schematic based on Issarti et al. (2021); **(e)** hexahedral mesh, author-created schematic based on Issarti et al. (2021); **(f)** tetrahedral mesh, author-created schematic based on Abdelhalim et al. (2021); **(g)** air-puff loading, author-created schematic based on Bronte-Ciriza et al. (2021); **(h)** stress contour, author-created schematic based on Issarti et al. (2021).

### 2.1 Geometry reconstruction

Finite element accuracy starts with geometry. Modern workflows use OCT or Scheimpflug-based systems such as Pentacam to capture layer thicknesses and surface curvatures (Figure 3a). Automated algorithms extract patient-specific contours, thicknesses, and vertex coordinates. Reconstruction techniques now reach sub-micron resolution and map stromal fiber orientation directly (Zvietcovich and Larin, 2022). Complementary use of multiple modalities, including OCT for high axial resolution and Pentacam for full anterior-segment topography has become standard practice to minimize reconstruction artifacts.

The resulting personalized model integrates individual anatomy with material properties. Iterative optimization routinely matches clinical data to within 95% and keeps displacement errors below 0.001 mm (Meng et al., 2022; Muench et al., 2022). Local refinements further align predicted ectatic shapes with follow-up observations (Rahmati et al., 2021). Clinical examples demonstrate clear biomechanical relevance: for LASIK procedures under physiological intraocular pressure (IOP = 15 mmHg), each additional 50 μm of residual central corneal thickness yields an approximately 12% reduction in postoperative peak von Mises stress, and this association shows a strong linear relationship (R^2^ = 0.89). This quantitative finding is explicitly derived from Wang et al. (2022), Figure 4, and is specific to LASIK rather than all refractive surgery modalities (e.g., SMILE, PRK) (Wang et al., 2022). These patient-specific geometries have proven especially powerful in cases of asymmetric keratoconus, where generic models fail to capture focal stress concentrations.

In the early stages of corneal biomechanical simulation, limited by computational resources and clinical measurement precision, geometric models were often simplified into rotationally symmetric structures based on idealized spherical or paraboloid surfaces (de la Caridad Núñez-Chongo et al., 2022; De La Hoz et al., 2024). Such “average geometry” models can qualitatively analyze the overall displacement under intraocular pressure (IOP) loading at a macroscopic level, but they fail to meet the clinical demands of precision medicine due to neglecting the non-uniform transition at the limbus, the asymmetry of the astigmatic axis, and the remarkable inter-individual differences in thickness distribution. With the innovation of three-dimensional tomography (Pentacam) and high-resolution OCT technologies, the modeling approach has transitioned from using generic idealized shapes to patient-specific anatomical reconstructions (Issarti et al., 2021; Wang et al., 2022). By extracting thousands of clinical spatial scatter points and using non-uniform rational B-splines (NURBS) or radial basis function (RBF) interpolation algorithms, researchers have reconstructed refined curved surfaces containing the complex topological relationships of the anterior surface, posterior surface, and limbus. However, such image-based “morphological reconstruction” is only the first step; the core challenge lies in transitioning from morphological reconstruction to biomechanical back-mapping.

The corneal geometric morphology obtained by clinical measurement is actually the equilibrium configuration of the tissue under IOP loading, rather than its mechanical initial state. Directly taking the clinically measured geometry as the initial input of the finite element mesh for secondary loading will lead to repeated superposition of geometric stiffness, causing the simulated cornea to exhibit artificially elevated stiffness and further resulting in a stress prediction deviation of approximately 10%–15%. This 10%–15% deviation is not a speculative consensus estimate, but a quantitatively validated benchmark from two specific finite element studies, i.e. Elsheikh et al. first identified that ignoring the zero-pressure (stress-free) configuration systematically overestimates corneal stiffness, and Ariza-Gracia et al. confirmed this translates to a 10%–15% error in von Mises stress prediction across 130 patient-specific models (healthy, keratoconic and post-LASIK corneas) under physiological intraocular pressure (IOP = 15 mmHg) (Elsheikh et al., 2013; Ariza-Gracia et al., 2016). To correct this physical inconsistency, the inverse finite element method (Inverse FEA) has become the core approach for constructing high-fidelity models. By establishing an objective function and performing multiple iterative optimizations in the numerical space, this algorithm inversely derives the clinically measured deformed morphology back to the “Zero-Pressure State” when IOP is zero. This biomechanical back-mapping strategy can initialize the stress field in the model, making the simulated preoperative equilibrium state physically consistent with the patient’s actual clinical images. This is critical for predicting the stress redistribution caused by tissue resection in refractive surgery and postoperative corneal morphological evolution (Redaelli et al., 2024).

Once the physical coordinate system is established, meshing the spatial discretization strategy directly determines the model’s ability to capture complex stress gradients. Since the cornea is a thin shell-continuum composite structure, traditional single-layer shell elements or coarse tetrahedral meshes are highly prone to shear locking or numerical oscillation when dealing with surgical cutting edges or gas impact loads. To accurately reproduce the mechanical heterogeneity of the corneal stroma along the depth direction, current advanced models generally adopt hybrid eight-node hexahedral elements (e.g., C3D8H) and set at least 3 to 5 discrete layers through the corneal thickness (Chen et al., 2024; Li et al., 2024). This hierarchical modeling method can not only support the precise assignment of fiber lamination angles in the subsequent anisotropic constitutive model but also accurately capture the differentiated strain responses of the anterior and posterior stromal layers under cutting pressure at the numerical level. In addition, to simulate scenarios involving interfacial mechanical interactions such as corneal transplantation or SMILE surgery, geometric modeling must also accurately restore the micro-gaps and contact characteristics between the donor and recipient, as well as between the corneal cap and the stromal bed (Joo et al., 2021; Li et al., 2021; Chen et al., 2024). This comprehensive mapping from macroscopic geometry to microscopic contact boundaries improves the finite element model from a mere static geometric replica of the patient’s eye into a dynamic digital twin with complete mechanical evolution capabilities, providing a robust biomechanical basis for the simulation of subsequent complex clinical scenarios.

### 2.2 Meshing

Mesh type and density balance accuracy against computation time. Hexahedral elements converge quickly and are efficient for regular geometries, while tetrahedral meshes handle irregular pathology or surgical incisions more flexibly (Table 1). For healthy corneas, central element sizes of 0.1–0.2 mm and peripheral sizes of 0.3–0.4 mm are typical; incised or diseased zones need local refinement to 0.05–0.08 mm. Grid-convergence-index checks and energy-norm monitoring keep errors below 5% (Joo et al., 2021; Li et al., 2021; Osmers et al., 2021; Rahmati et al., 2021; Muench et al., 2022; Ramasubramanian et al., 2022; Nambiar et al., 2023; Fan et al., 2024). Many recent studies now employ hybrid meshing combining hexahedral cores with tetrahedral transition layers to achieve both speed and geometric fidelity, particularly around surgical cuts or ectatic regions. Adaptive meshing is recommended whenever large deformations or strong nonlinearity appear (Ramasubramanian et al., 2022; Fan et al., 2024).

**TABLE 1:** Guidelines for mesh partitioning strategy and convergence of corneal finite element models.

Dimension	Hexahedral mesh	Tetrahedral mesh	General guidelines and convergence analysis
Core Features	High precision, efficient convergence, regular element shapes	Good adaptability and flexibility for complex geometry	-
Computational Accuracy	High (GCI <1%, deviation from experiment <5%)	Moderate, requires local mesh refinement compensation	Density setting principles: match the geometric and mechanical gradients Healthy cornea: central 0.1–0.2 mm, peripheral 0.3–0.4 mm Pathological/surgical area: locally refine to 0.05–0.08 mm
Convergence Efficiency	High (30%–40% fewer iterations)	Lower (30%–50% more elements, 2–3 times longer)	Convergence verification: GCI convergence analysis, energy norm convergence
Geometric Adaptability	Poorly adaptable to irregular geometries	Good, can automatically fill complex areas	Boundary layer mesh: surfaces with high curvature require prism elements for transition
Typical Application Scenarios	Generic healthy cornea model, routine surgical simulation	Personalized/pathological models, transplant surgery simulation	-
References	Issarti et al. (2021), Xiao et al. (2022), Zvietcovich and Larin (2022)	Osmers et al. (2021), Meng et al. (2022), Ramasubramanian et al. (2022), Fan et al. (2024)	Joo et al. (2021), Li et al. (2021), Meng et al. (2022), Ramasubramanian et al. (2022), Xiao et al. (2022), Zvietcovich and Larin (2022)

### 2.3 Boundary and loading conditions

*In vivo*, the cornea experiences intraocular pressure (typically 10–21 mmHg), extraocular muscle tension, and contact loads from instruments or lenses (Osmers et al., 2021; Nambiar et al., 2023; Fan et al., 2024). Clinical images show the cornea in its pressurized state, not its stress-free reference shape. Iterative correction using inverse algorithms or energy-based methods now recovers the unloaded geometry reliably, eliminating systematic errors in stress calculations (Muench et al., 2022; Li et al., 2025). In practice, these inverse methods are applied iteratively until the simulated pressurized shape matches the clinical image within sub-micron tolerance, a step that has substantially improved.

For large-deformation scenarios such as orthokeratology or surgical contact, penalty-function contact algorithms handle interface mechanics (Wang S. et al., 2023; Hsiao et al., 2024). Fluid-structure interaction methods are increasingly used to simulate air-puff tonometry or aqueous-humor flow, moving the field closer to true “digital twin” behavior (Safdel and Hassani, 2021; de la Caridad Núñez-Chongo et al., 2022; Redaelli et al., 2022).

#### 2.3.1 Corneoscleral limbus-sclera attachment modeling

The modeling strategy for the corneoscleral limbus-sclera interface is a foundational decision that strongly governs peripheral corneal stress distribution, incision biomechanics, and postoperative ectasia risk prediction. Early numerical models adopted fully clamped boundary conditions by rigidly constraining all limbal nodes to zero displacement and rotation, treating the corneoscleral junction as an immovable clamp (Elsheikh and Wang, 2007). This approach minimizes computational cost but artificially overestimates corneal stiffness by 15%–20% and suppresses physiological peripheral deformation, overpredicting central corneal stress while underestimating peri-limbal stress concentration; it is therefore unsuitable for simulations involving peripheral incisions, limbal relaxing incisions, or corneal periphery refractive surgery.

A simplified yet physiologically improved alternative is the tangential sliding or pivoting boundary condition, which omits explicit scleral geometry but allows limbal nodes free tangential sliding along the limbal circumference while restricting radial displacement (Gómez et al., 2024). This design captures the natural rotational flexibility of the limbus without increasing model complexity, reducing stress prediction error by approximately 8% relative to fixed boundaries. However, it cannot reproduce load transfer between the cornea and sclera or capture regional stiffness gradients at the limbus.

The most anatomically realistic framework is explicit scleral modeling, which integrates the sclera as a distinct structural domain with independent geometry and material properties. Two standard geometric configurations are widely adopted: a partial scleral ring (2–5 mm wide circumferential scleral strip) that balances accuracy and efficiency and suffices for most corneal biomechanics and refractive surgery simulations (Elsheikh and Wang, 2007; Issarti et al., 2021), and a full ocular globe (complete cornea plus continuous scleral shell) that is required for whole-eye biomechanics, glaucoma, or air-puff tonometry simulations (Issarti et al., 2021; De La Hoz et al., 2024).

The sclera is most commonly defined as a transversely isotropic or anisotropic hyperelastic material using Ogden, Mooney-Rivlin, or Holzapfel-Gasser-Ogden (HGO) models, with a Young’s modulus 3–10 times higher than that of the central cornea (Ji et al., 2023; Ji et al., 2026). Sensitivity studies confirm that corneal stress and apical displacement predictions are highly sensitive to scleral stiffness: a 50% increase in scleral modulus reduces central corneal apical displacement by approximately 12% under physiological IOP (15 mmHg) and shifts peak stress toward the limbus (Issarti et al., 2021). Overly rigid scleral assumptions falsely stabilize postoperative deformation, while overly compliant sclera models overestimate ectasia risk, highlighting the clinical relevance of appropriate scleral mechanical representation.

### 2.4 Definition of material models

Constitutive laws determine simulation fidelity. Early models used isotropic linear elasticity for tiny deformations. Hyperelastic formulations (e.g., Ogden or Mooney-Rivlin) later captured the cornea’s low-strain compliance and high-strain stiffening (Wang et al., 2023a; Fan et al., 2024). Viscoelastic models added time-dependent relaxation and creep, which matter for long-term CXL stability (Fontanella et al., 2021; Rahmati et al., 2021). Current anisotropic fiber-reinforced models reproduce direction-dependent stiffness and are essential for keratoconus progression, surgical cuts, and pressure fluctuations (Ji et al., 2023; Fantaci et al., 2024a; Kory et al., 2024). Recent implementations incorporate depth-dependent fiber dispersion and crimping, allowing more accurate representation of the anterior stroma’s higher stiffness compared with posterior layers. When local fiber disruption occurs, stress redistributes dramatically, offering quantitative markers for clinical diagnosis (Gómez et al., 2021; Nambiar et al., 2022; Gómez et al., 2024; Lin et al., 2024) (Figure 4).

**FIGURE 4:**
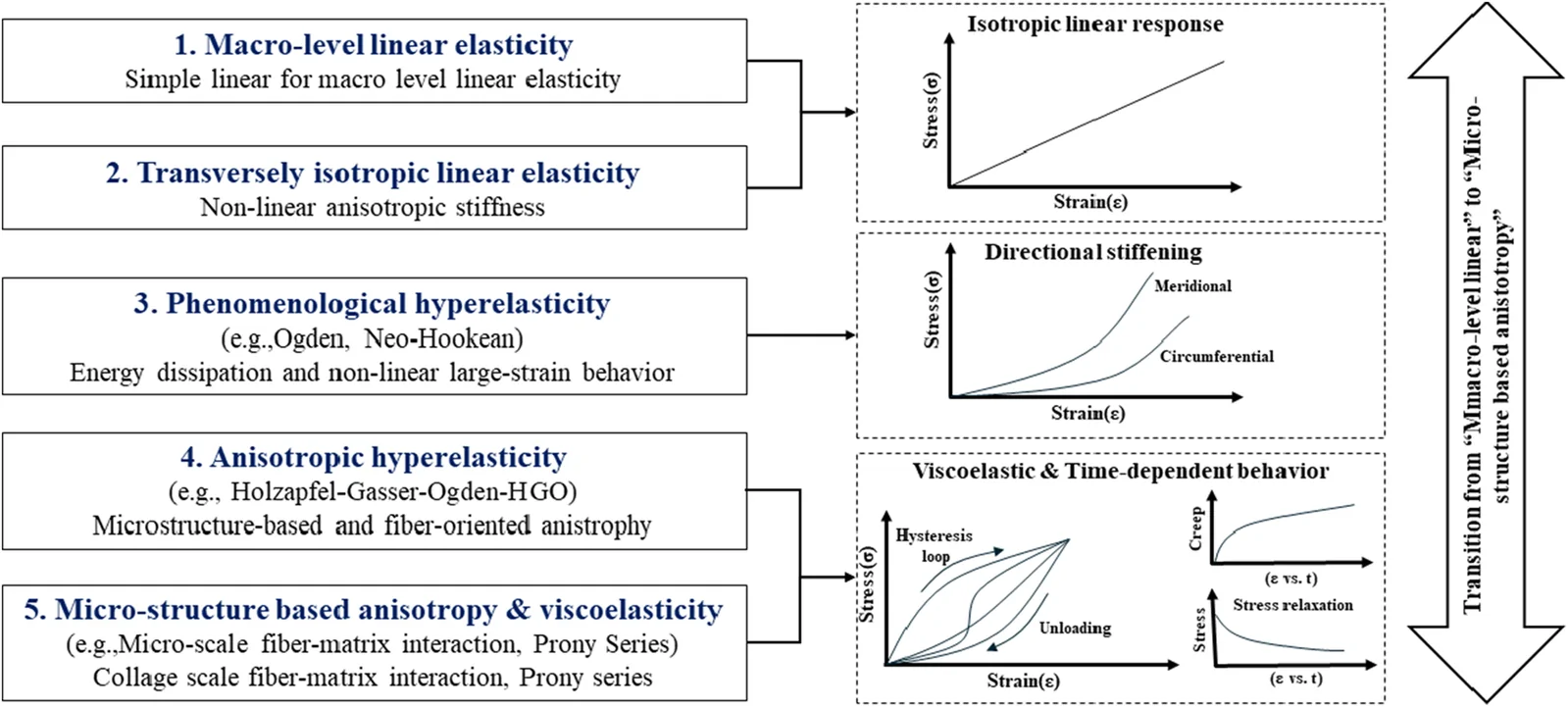
Evolution of corneal constitutive models. Original figure created by the authors based on the scientific information reported in Mahdian et al. (2021), de la Caridad Núñez-Chongo et al. (2022), and Soloviev et al. (2024).

In finite element modeling of corneal biomechanics, constitutive model selection dictates simulation accuracy and bridges microscale pathology to macroscale mechanical response. Linear elastic models are obsolete, as they cannot replicate the cornea’s nonlinear J-shaped stress-strain behavior under physiological intraocular pressure (IOP). Modern frameworks adopt hyperelastic models (Ogden, Mooney-Rivlin) via strain-energy density functions to capture strain-stiffening, enabling reliable displacement prediction under physiological loading (Wang et al., 2023b; Fan et al., 2024).

As a fiber-reinforced composite with aligned collagen lamellae, the cornea requires anisotropic constitutive descriptions. The Holzapfel-Gasser-Ogden (HGO) model is the most widely adopted framework in current corneal FE modeling, embedding collagen orientation into strain-energy formulation to reproduce directional stiffness (central orthogonal fibers, limbal circumferential fibers) and capture meridional-circumferential mechanical asymmetry. Within the HGO framework, two fiber-distribution methods are widely used:

1. The first is the Generalized Structure Tensor (GST) approach. It uses a single structure tensor to represent average fiber orientation and dispersion, prioritizes computational efficiency (Fantaci et al., 2024b).
2. The second is the Angular Integration approach. It explicitly integrates over continuous fiber-direction distributions, delivers higher accuracy for dispersed fiber networks but with increased computational cost (Büchler et al., 2025).

To standardize material parameter selection for corneal finite element modeling, Table 2 summarizes the core mechanical properties across linear elastic, hyperelastic, anisotropic (HGO), and microstructural frameworks. It compiles reported parameter ranges, their derivation approaches (experimental calibration, inverse finite element analysis, or literature consensus), and corresponding references, providing a unified reference for model setup and reducing variability in simulation outcomes. Importantly, a recent study by González-Cabrero et al. demonstrated that material parameters estimated from uniaxial tensile tests versus inflation tests for the same HGO model are not directly interchangeable. For example, the fiber nonlinearity parameter *k*_2_ differed by nearly an order of magnitude (tensile tests gave a *k*_2_ value of roughly 24, whereas inflation tests yielded a value of about 223), despite both fits achieving excellent correlation (*R* ^2^ = 0.99). This finding underscores the critical need for Table 2: by clearly documenting the derivation approach (e.g., tensile vs. inflation vs. biaxial) for each reported parameter, the table helps modelers select values that are consistent with their specific experimental or clinical context, thereby avoiding erroneous parameter transfer across different testing modalities. (González-Cabrero et al., 2026).

**TABLE 2:** Summary table of corneal material properties.

Property category	Parameter	Reported range	Parameter derivation approach	Reference
Linear Elastic	Young’s modulus (E)	0.2–10 MPa	Uniaxial tensile testing; inverse FEA	Karimi et al. (2018)
	Poisson’s ratio (ν)	0.45–0.49	Literature consensus; incompressibility assumption	Kling et al. (2014)
Hyperelastic (Mooney-Rivlin)	C_10_, C_01_	−101.533, 107.793 kPa	Fitting to Corvis air-puff deformation	Karimi et al. (2018)
Hyperelastic (Ogden)	μ_1_, α_1_	0.01–0.1 MPa, 8–12	Biaxial testing; inverse FEA	Wang et al. (2023b)
Anisotropic Hyperelastic (HGO)	k_1_ (anterior/posterior)	3.46/1.10 MPa	Depth-dependent human corneal tensile testing	Büchler et al. (2025)
	k_2_	21.0	Experimental fitting of strain-stiffening	Büchler et al. (2025)
	Fiber dispersion (κ)	0.05–0.35	X-ray scattering; inverse FEA (healthy/keratoconus)	Fantaci et al. (2024b)
	In-plane fiber parameter (a)	0 (anterior) → 5 (posterior)	Lamellar orientation gradient; X-ray validation	Büchler et al. (2025)
	Out-of-plane parameter (b)	2.5	Fiber angular distribution fitting	Büchler et al. (2025)
Microstructural (Collagen Network)	Fibril stiffness (Dբ)	0.009 N	Homogenized corneal stromal mechanics	Pandolfi et al. (2019)
Microstructural (Cross-link)	Inter/intra-fibril stiffness	0.0072/0.0045 N	Molecular bond mechanics; truss model calibration	Pandolfi et al. (2019)

As research advances toward the mesoscopic scale, phenomenological macroscopic constitutive relations fail to capture the mechanical instability underlying complex pathologies such as keratoconus. Multiscale modeling, referring to discrete-to-continuum upscaling and microstructural-macroscopic coupled frameworks, has emerged as a rigorous approach to link microscale collagen network deterioration to macroscale corneal deformation and ectasia. Unlike conventional models that simplify the cornea into a homogeneous medium, advanced multiscale models explicitly introduce representative volume elements (RVEs) or discrete microstructural truss networks to resolve fine-scale mechanisms. Two clinically relevant frameworks have been validated for keratoconus:

1. Discrete-to-continuum multiscale modeling builds a discrete network of collagen lamellae and proteoglycan cross-links, then upscales microstructural force balance to macroscopic continuum equations via asymptotic homogenization, enabling quantitative prediction of conical protrusion (Kory et al., 2024).
2. Microstructural fibril-cross-link modeling represents the cornea as a discrete trusswork of collagen fibrils and inter-fibrillar cross-links, explicitly simulating fibril sliding, cross-link rupture, and fiber crimp loss. These microscale failures are converted into macroscopic stiffness degradation via strain energy homogenization to macroscopic stiffness degradation via strain energy homogenization (Pandolfi et al., 2019).

This framework abandons the homogeneous assumption and uses energy bridging/homogenization schemes to directly map microstructural evolution (fiber disorganization, cross-link loss) to macroscopic mechanical weakening and geometric expansion in keratoconus (Pandolfi et al., 2019; Fantaci et al., 2024a; Kory et al., 2024).

Within this framework, the physical meaning of the fiber dispersion parameter *κ* is elevated: it is not merely a statistical distribution index, but a quantitative indicator characterizing the transition of collagen arrangement from “highly ordered” to “randomly disordered” under pathological conditions. Experimentally calibrated *κ* values show clear stratification with keratoconus severity: in healthy human corneas, *κ* ranges from 0.05–0.15, representing tightly aligned collagen fibrils (Nambiar et al., 2022); in mild keratoconus (Belin ABCD grade A1-A2), *κ* increases to 0.15–0.25; in moderate keratoconus (grade A3-A4), κ rises to 0.25–0.35; and in advanced keratoconus (grade A4/B4, *K*_max_ > 60 D), *κ* exceeds 0.35 (Fantaci et al., 2024b; Pandolfi and De Bellis, 2024). As *κ* increases, the internal stress distribution undergoes dramatic reorganization. The circumferential tension originally borne by fibers in specific directions is converted into local shear stress concentration, which constitutes the mechanical cause of localized thinning and conical protrusion in keratoconic lesions (Gómez et al., 2021; Nambiar et al., 2022; Gómez et al., 2024; Lin et al., 2024).

Through multiscale coupling, researchers can more clearly observe that CXL treatment does not simply increase the overall modulus; rather, it restricts relative sliding between fibers by increasing cross-link density at the microscale, thereby restoring deformation resistance macroscopically.

In addition to spatial heterogeneity, the cornea being a biological tissue with high water content exhibits time-dependent viscoelastic behavior, which also affects the validity of clinical measurements. Traditional quasi-static models often overestimate the instantaneous stiffness of the material when simulating high-speed air-puff loads such as those in corneal visualization Scheimpflug technology (Corvis ST), due to the neglect of dynamic damping effects. Therefore, embedding viscoelastic terms based on the generalized Maxwell theory into multiscale constitutive models has become a standard configuration for simulating dynamic mechanical responses (Fontanella et al., 2021; Rahmati et al., 2021).

Viscoelastic parameters, such as relaxation time and viscosity coefficient, govern not only the stress diffusion rate within the tissue but also its energy dissipation capacity. In clinical practice, this time-dependent mechanical behavior explains why keratoconic corneas display a longer recovery cycle after transient impact, providing a more sensitive biomechanical marker for distinguishing subclinical keratoconus than purely geometrical indices (Rahmati et al., 2021).

At present, the development of constitutive models is evolving toward biochemical-mechanical bidirectional coupling. Such high-order multiscale models attempt to improve biochemical processes such as collagen degradation induced by matrix metalloproteinases (MMPs) into time-evolution equations for material parameters within the constitutive framework. This means that the finite element model will no longer be a static simulation tool, but a adaptive computational framework whose parameters dynamically evolve with disease progression and time.

This in-depth biomechanical back-mapping enables researchers to predict the corneal mechanical state years or even decades in advance, providing a genuine quantitative early-warning mechanism for evaluating the long-term safety of refractive surgery. Such a paradigm shift from macroscopic phenomenological description to microscopic mechanistic reconstruction has not only greatly improved simulation fidelity but also provided an irreplaceable theoretical tool for deciphering the pathogenesis of complex corneal diseases (Ji et al., 2023; De La Hoz et al., 2024; Fantaci et al., 2024a; Kory et al., 2024).

### 2.5 Multi-physics modeling

Coupling structural mechanics with fluid or thermal fields captures real-world interactions. Air-puff simulations now reproduce the three classic deformation phases: initial deformation, applanation, and maximum concavity yielding quantitative biomarkers such as inverse radius and deflection area (Li et al., 2023). Aqueous-humor computational fluid dynamics (CFD) models reveal pressure-driven flow patterns and temperature distributions; natural convection reverses when ambient temperature exceeds body temperature, a finding useful for assessing laser-induced thermal risk (Bhandari, 2021; Cai et al., 2022). Recent studies have further integrated thermal-structural coupling during excimer laser ablation, showing that transient temperature rises can temporarily reduce stromal modulus by up to 20%, an effect that must be accounted for in long-term stability predictions.

## 3 Typical applications of FE in corneal biomechanics

As finite element models transition from theoretical frameworks to clinical decision support, their core value lies in quantitative evaluation and prognostic simulation of corneal biomechanical changes under different surgical interventions. As the most widely applied clinical scenario, corneal refractive surgery can essentially be interpreted as a biomechanical optimization process of “how to maximally preserve stromal integrity”.

Early surface ablation procedures, such as PRK, exert mechanical responses mainly on the ablation of the anterior-most Bowman’s membrane and anterior stroma. Numerical simulation studies reveal that although PRK avoids structural damage caused by flap creation, epithelial remodeling and keratocyte apoptosis during postoperative healing may disturb long-term mechanical stability, and thermal damage induced by laser energy also forms a local modulus gradient in the superficial stroma (Abdelhalim et al., 2021; De Stefano et al., 2022). In contrast, the corneal flap introduced in LASIK exerts a profound impact on biomechanical integrity by disrupting anterior stromal load-bearing layers. Small incision lenticule extraction (SMILE) represents a middle ground, preserving the anterior cap while removing lenticules through small incisions. Direct quantitative comparisons from patient-specific finite element simulations Table 3 demonstrate consistent biomechanical differences among the three procedures under physiological intraocular pressure, with clear correlations between residual stromal bed thickness, peak stress elevation, and clinical risk.

**TABLE 3:** Quantitative comparison of finite element-predicted biomechanical outcomes among PRK, LASIK, and SMILE.

Reference	# of patients (if any)	Geometry	Constitutive law	Element type/Software	Key parameters	Main finding
Wang et al. (2022)	N = 1 (representative)	Patient-specific 3D	Hyperelastic anisotropic (HGO)	Not specified/ABAQUS	15–31 mmHg (multiple levels); RSB thickness: 311–353 μm; myopia correction: −3.0 D and −6.0 D	SMILE results in lower corneal displacement and von Mises stress than LASIK under identical conditions, with linear IOP-dependent increases, and offers better biomechanical stability and reduced ectasia risk
Büchler et al. (2025)	N = 30	Patient-specific 3D	Depth-dependent anisotropic HGO	C3D8R/ABAQUS	IOP = 15 mmHg; myopia correction: −7.00 to −1.25 D	PRK avoids flap-induced interface stress; postoperative peak stress increases by only +28% vs. baseline (vs. +53% for LASIK); stress distribution remains uniform in the anterior stroma
Fantaci et al. (2026)	N = 58	Patient-specific 3D	Patient-calibrated anisotropic hyperelastic	C3D10M/ABAQUS 2022	Patient-specific wIOP (range 8.93–16.1 mmHg); lenticule thickness: 64–140 μm (central ablation depth, CAD); myopia correction: −1.5 to −6.25 D (observed range)	Patient-specific material properties and IOP were incorporated into FE simulations for the first time, yielding a mean postoperative dioptric error of +0.40 ± 0.30 D. Mechanically, SMILE induced the largest increases in RSB stress (+68.2%) and strain (+48.8%), whereas PRK was the least invasive

With the rise of SMILE, finite element simulation has provided critical mechanical evidence to verify its minimally invasive nature. Comparative studies demonstrate that because SMILE requires only a 0–2 mm side incision, the anterior corneal cap the stiffest stromal layer, bearing approximately 40%–50% of the mechanical load remains intact. Mechanical calculations indicate that under the same equivalent refractive ablation volume, corneal integrity parameters after SMILE are significantly superior to those after LASIK, with deformation resistance and postoperative peak stress closer to the natural physiological state. This advantage is particularly prominent in high-myopia cases with large ablation volumes, where the model-predicted corneal ectasia risk threshold is extended due to the preservation of the cap layer (De Stefano et al., 2022; Wang et al., 2022; Fang et al., 2023; Montanino et al., 2023). Current clinical simulations have gone beyond mere geometric ablation prediction, and begun to deeply integrate patient-specific constitutive parameters. Using the initial stress field retrieved by the inverse finite element method, integrated evaluation of postoperative contrast sensitivity, astigmatism vectors, and biomechanical stability (e.g., Stress-Strain Index (SSI) evolution) has been realized (Figure 5).

**FIGURE 5:**
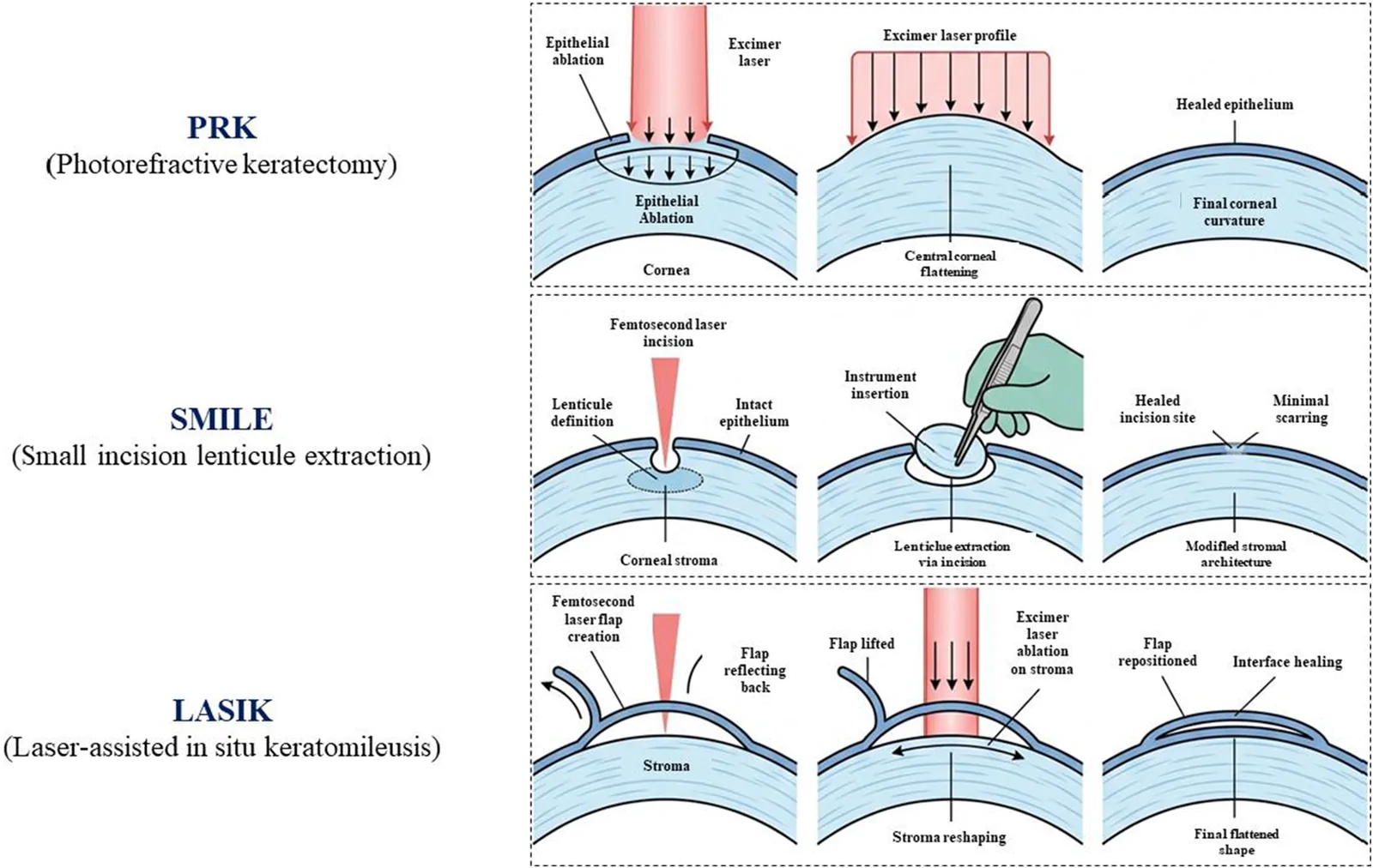
Comparison of refractive surgery techniques: corneal structural impact. Original schematic created by the authors based on the scientific information reported in Du et al. (2021) and Fantaci et al. (2025).

In addition to refractive surgery, finite element modeling has been extensively applied to keratoconus/corneal cross-linking (CXL), corneal transplantation, and orthokeratology. Table 4 summarizes representative studies in these domains, highlighting key modeling features and biomechanical findings.

**TABLE 4:** Summary of finite element studies on keratoconus/CXL, keratoconus/ICRS, corneal transplantation, and orthokeratology.

Application domain	Reference	Key modeling features	Main biomechanical findings
Keratoconus/CXL	Fantaci et al. (2024b)	Hyperelastic anisotropic HGO model with reduced fiber stiffness to mimic pathological degradation	Regional stiffness of keratoconic cone reduces by 41%–60%; Material degradation induces progressive corneal bulging and thinning, reproducing typical keratoconus phenotypes
	Roy and Dupps (2011)	Patient-specific FE model with graded weakening; simulated CXL with 200–300 µm depth	Increasing CXL depth from 200 to 300 µm enhances corneal flattening by 24%–34%; reduces higher-order aberrations by 22%–31%
	Pandolfi et al. (2019)	Microstructural fibril-cross-link truss model with two types of cross-links	Weakening of inter-fibril cross-links dominates corneal bulging and excessive deformation in keratoconus
	Frigelli et al. (2025)	Patient-specific FEM, depth-dependent HGO model, KC weakening (57%), CXL stiffening via KCXL -scaling; customized: 14 J/cm2 on cone (1.33×)	Customized CXL flattens more (ΔKmax-t −2.28 D vs. −0.81 D); predictions correlate (R2 = 0.59); sensitive to IOP and KC weakening (up to 20% variation)
	Wang and Chester (2021)	Multi-physics (diffusion + UV + reaction + mechanics); Abaqus UEL; anisotropic HGO; stiffness ∝ cross-linking degree ξ	Reproduces CXL stiffening; matches IOP-displacement experiments; cross-linked cornea deforms less; enables 3D personalized planning
Keratoconus/ICRS	Kling and Marcos (2013)	2D axisymmetric FEM; hyperelastic cornea (Mooney-Rivlin); triangular and hexagonal ICRS cross-sections; varied ring height (150–350 μm) and optical zone (4.4–6.6 mm); simulated central keratoconus via local stiffness reduction	ICRS height and optical zone significantly affect corneal flattening (corneal power reduction: −4.08 to −17.7 D in healthy, −3.31 to −20.5 D in KC); triangular ICRS more effective than hexagonal. Refractive change contributions: anterior surface −75.4% (flattening), posterior surface +12.3% (steepening, counteracts flattening), apex shift −13.4% (enhances flattening)
	Lago et al. (2015)	First patient-specific 3D FEM for ICRS in KC; geometry from Pentacam; hyperelastic Ogden model (*in vivo* calibrated); simulated ring placement at clinical depth/angle; validated against 7 patient outcomes	Simulation predicted corneal flattening consistent with clinical outcomes; mean curvature radius error ∼0.74 mm; demonstrates potential for patient-specific surgical planning and ring design optimization
	Flecha-Lescún et al. (2018)	Semi-automatic template-based 3D FE model including the cornea, limbus, and sclera; recovery of the stress-free configuration and physiological IOP prestressing; incision elements removed and expanded using a fluid-cavity procedure; solid circular PMMA ICRS with frictionless contact; 33 full-factorial analysis and ray-tracing-based optical assessment	Explicit simulation of the implantation procedure avoided the artificial presurgical weakening associated with a predefined tunnel gap and allowed free 3D motion of the implanted ring segments. Stromal insertion depth was the most influential parameter for spherical and cylindrical changes; deeper implantation altered corneal bending and local stress redistribution, while the effect on cone-region stress depended on the spatial location of the weakened tissue
	Ariza-Gracia et al. (2020)	Clinically calibrated optomechanical FE framework; 3D quarter-symmetry model for ring segments and 2D axisymmetric models for continuous rings; Yeoh isotropic hyperelastic corneal stroma; comparison of tunnel and pocket implantation and generic versus commercial ring geometries; geometric ray tracing and spatially resolved corneal strengthening factor; calibration and population-level evaluation using clinical MyoRing data	The model predicted the population-level change in spherical equivalent with an overall error below 0.6 D. Implant size and diameter were major determinants of refractive correction, whereas the influence of implantation depth depended on surgical technique. Central stress changes were limited, supporting a localized bulkening and kinematic mechanism rather than global corneal stiffening; continuous rings implanted in pockets produced greater refractive correction than tunnel-based implants
	García de Oteyza et al. (2021)	2D FEM with transversely isotropic cornea (epithelium + anterior/posterior stroma); simulated asymmetric ICRS with combined thickness (150–300 μm) and base-width (600–800 μm) variation; compared isolated thickness vs. width effects	Asymmetric ICRS balanced flattening across ring arc; thickness dominated corneal flattening (∼0.13 D/μm) over width (∼0.04 D/μm); combined variation better preserved surface homogeneity for asymmetric KC phenotypes
	Bahramizadeh-Sajadi et al. (2024)	Automated axisymmetric FE pipeline; Yeoh hyperelastic cornea; 300 configurations (20 ring designs × 3 diameters × 5 sizes); fixed 75% depth; varied cross-sectional shape, vertical/horizontal dimensions, and implantation diameter (5–6 mm)	Vertical ring dimension accounts for ∼84% of curvature change; cross-sectional shape contributes ∼13%; horizontal dimension and ring diameter have minor effects; vertical rings induce higher contact stress
	García de Oteyza et al. (2023)	3D shell FEM (healthy and KC cornea); hyperelastic isotropic (Yeoh); compared symmetric vs. progressive asymmetric ICRS (variable thickness 150–300 μm, base width 600–800 μm); assessed sagittal curvature and Zernike aberrations	Asymmetric ICRS better corrected vertical coma (−3.42 vs. −3.13 μm) and defocus (2.90 vs. 2.75 μm) than symmetric design; thickness variation primarily addressed horizontal coma and oblique trefoil; phenotype-specific ICRS design enables tailored aberration correction
Corneal Transplantation (PK, DALK, DSEK)	Li et al. (2021), Chen et al. (2024)	3D FE model with suture mechanics and graft-host interface	Sutures cause extreme asymmetric limbal stress, leading to postoperative irregular astigmatism in PK
	Joo et al. (2021), Opfermann et al. (2024)	Simulation of appositional pressure in DSEK/DMEK	Optimal air-injection pressure prevents graft dislocation without damaging endothelial cells
Orthokeratology (OK Lens)	Ramasubramanian et al. (2022), Wu et al. (2024)	Fluid-structure interaction (FSI) with tear-film hydrodynamics	Reverse-geometry lens creates central positive pressure and mid-peripheral negative suction, inducing epithelial migration and stromal reshaping
	Aguilella-Arzo and Compañ (2023), Meng et al. (2025)	Coupled oxygen diffusion and viscoelastic response	Predicts diurnal thickness fluctuations and cumulative stiffness changes after long-term OK lens wear

Collectively, these FE studies have elucidated the mechanical underpinnings of keratoconus progression, quantified the stiffening effects of CXL, optimized suture strategies in transplantation, and revealed the tear-film pressure mechanisms in orthokeratology. The quantitative biomarkers derived from these models—such as stress concentration factors, stiffness gradients, and pressure distributions—provide valuable guidance for personalized clinical management. Figure 6 systematically demonstrates the multidimensional capabilities of finite element models in addressing complex clinical corneal problems. The upper part of the figure shows the stress distribution differences between a healthy eye and a KC eye under equivalent intraocular pressure, clearly revealing the stress concentration phenomenon in the lesioned area (apex). The upper right further illustrates the gradient-reinforced zone of physical properties formed within the stromal layer after CXL. The middle and lower parts of the figure respectively map the evolution trajectory of the stress-strain index (SSI) derived from Corvis ST simulations, as well as the dynamic induction effect of the tear film pressure field on the corneal surface during OK lens wear. This set of illustrations intuitively proves that finite element simulation can bridge the scale gap from microscopic material reinforcement to macroscopic morphological remodeling, providing visualized mechanical evidence for precise clinical diagnosis and treatment (Bagheri et al., 2021; Xiao et al., 2022; Sanchez-Real et al., 2023).

**FIGURE 6:**
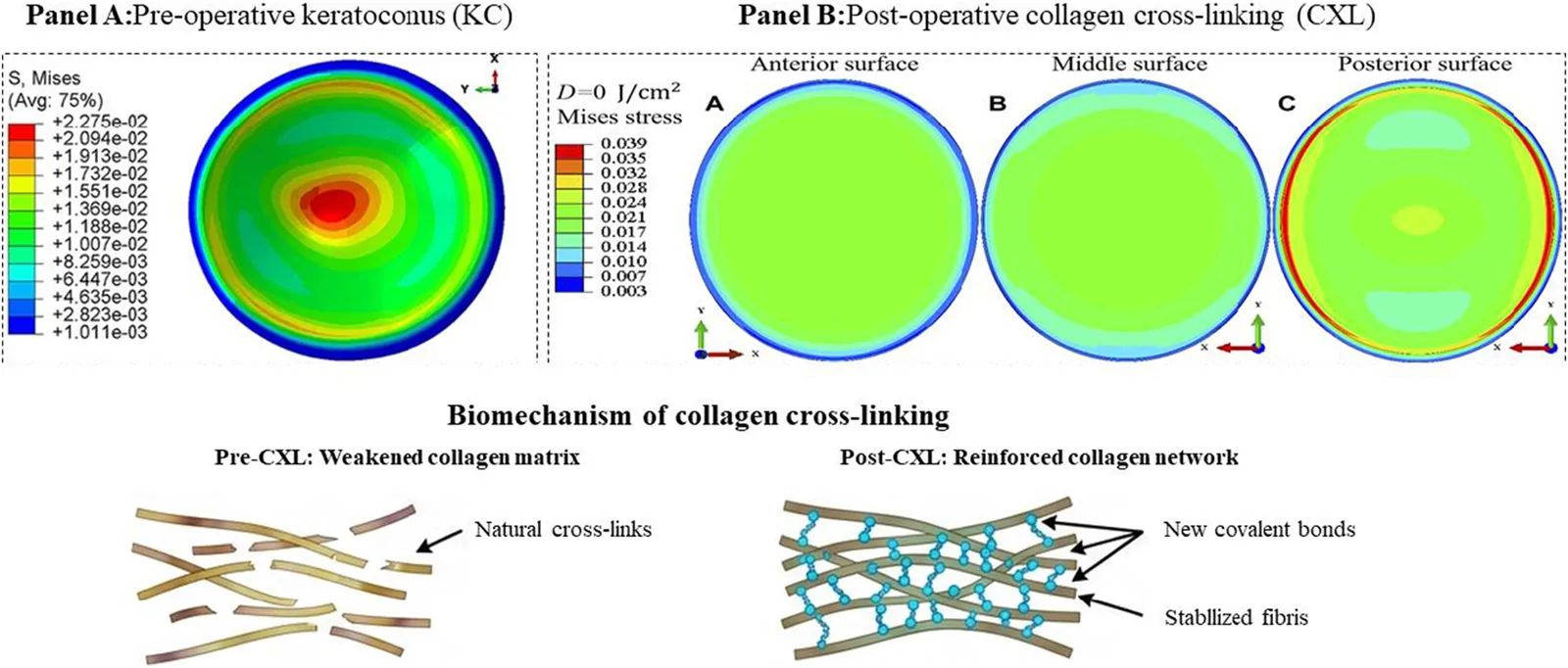
Professional consolidated scientific result map: **(A)** Pre-operative keratoconus (KC), adapted from Bagheri et al. (2021) under a Creative Commons Attribution (CC BY) license, with cropping and resizing only; **(B)** Post-operative collagen cross-linking (CXL), adapted from Xiao et al. (2022) under a Creative Commons Attribution (CC BY) license, with cropping and resizing only. The collagen cross-linking mechanism diagrams below were created by the authors based on the scientific information reported in Sanchez-Real et al. (2023).

In addition to CXL, finite element modeling has been applied to optimize intracorneal ring segment (ICRS) implantation for keratoconus management. Distinct modeling strategies have been proposed to represent either the implantation procedure itself or the resulting postoperative optomechanical response. Flecha-Lescún et al. developed a semi-automatic, template-based three-dimensional FE methodology in which the physiological prestressed state was first established, followed by removal and pressurization-assisted expansion of the incision elements and insertion of solid ICRS with explicit ring–stroma contact. This approach avoided introducing a pre-existing tunnel gap in the presurgical configuration and allowed three-dimensional rigid-body motion of the implanted segments. A 3^3^ full-factorial analysis was subsequently used to evaluate the effects of stromal insertion depth, radial insertion position, and ring cross-sectional diameter on corneal mechanics and ray-traced optical outcomes (Flecha-Lescún et al., 2018).

Ariza-Gracia et al. subsequently adopted a complementary, clinically calibrated optomechanical framework. A three-dimensional quarter-symmetry model was used for ring segments, whereas axisymmetric models were employed for continuous rings, enabling efficient comparisons among segmental and continuous implants placed in intrastromal tunnels or pockets. The effects of implant geometry and position, postoperative ring rotation, intraocular-pressure variation, and keratoconic tissue weakening were systematically assessed. The model was calibrated using clinical MyoRing data and evaluated at the population level, while a spatially resolved corneal strengthening factor was introduced to distinguish local stress redistribution from global corneal stiffening. The results suggested that intrastromal implants primarily produce localized bulkening and redirect corneal kinematics rather than globally stiffening the cornea; moreover, the sensitivity to implantation depth depended on whether the implant was placed in a tunnel or a pocket (Ariza-Gracia et al., 2020). Both frameworks nevertheless employed idealized central or average corneal geometries and isotropic hyperelastic tissue descriptions. In addition, the template-based approach was restricted to predefined parameter combinations, whereas the axisymmetric formulation limited the assessment of asymmetric keratoconus and astigmatic changes, motivating the development of later patient-specific and more extensively parameterized models.

Subsequent studies increasingly incorporated patient-specific geometries, anisotropic constitutive laws, and broader design spaces. Bagheri et al. developed patient-specific FE models with anisotropic HGO material properties to evaluate the effects of ring design parameters, including thickness, arc length, and implantation depth, as well as surgical technique, including tunnel and pocket implantation, on postoperative corneal biomechanics and keratometry (Bagheri et al., 2021). Their simulations demonstrated that ring thickness and implantation depth were major determinants of corneal flattening, with changes in implantation depth also affecting localized stress concentrations at the ring–tissue interface. Bahramizadeh-Sajadi et al. further extended parametric assessment of intrastromal ring design by systematically evaluating cross-sectional shape, vertical and horizontal dimensions, and implantation diameter (Bahramizadeh-Sajadi et al., 2024). Collectively, these studies illustrate the progression of ICRS modeling from procedural simulation and mechanism-oriented optomechanical analysis to patient-specific prediction and large-scale design optimization, supporting personalized ring selection and more informed surgical planning.

In summary, finite element analysis of the cornea in clinical settings is no longer confined to morphological simulation. Instead, by constructing a complete mechanical closed loop of “geometry-constitution-loading”, it reconstructs the causal chain between microscopic tissue damage and macroscopic functional degradation. From ablation optimization in refractive surgery and dynamic early warning of keratoconus to stress balance at transplantation interfaces, finite element models are intervening in clinical decision-making with unprecedented depth. The future challenge lies in translating these massive numerical results into interpretable and operable biomechanical indicators for clinicians, and achieving full-lifecycle precision management preoperatively, intraoperatively, and postoperatively through deep integration with artificial intelligence and digital twin technology.

## 4 Challenges and outlook

Despite considerable advances in corneal finite element analysis over the past two decades, a genuine bridge between laboratory research and real-time clinical diagnosis still requires in-depth technical reflection on the bottlenecks of model fidelity under dynamic conditions and computational efficiency.

A primary challenge lies in accurately characterizing FSI effects. Currently, the most widely used clinical instruments for *in vivo* corneal biomechanical assessment, such as Corvis ST and the ocular response analyzer (ORA), rely on high-frequency dynamic air-puff tests. However, the vast majority of existing finite element models still simplify this process as a pure solid mechanics problem, applying only a time-dependent pressure load on the anterior corneal surface while neglecting the damping effect of the incompressible aqueous humor inside the eye. The flow of aqueous humor and the resulting reverse pressure gradient during severe corneal deformation not only alter the corneal deformation amplitude but also profoundly affect the extraction of stress dissipation indices. Studies have shown that pure structural models tend to overestimate the macroscopic stiffness of the cornea due to the lack of fluid viscous damping, leading to significant discrepancies between model-predicted deformation and clinical observations (Safdel and Hassani, 2021; Redaelli et al., 2022; Yong et al., 2024).

From the interdisciplinary perspective of engineering and biology emphasized in journals such as Frontiers, the introduction of FSI models based on the arbitrary Lagrangian-Eulerian (ALE) method or smoothed particle hydrodynamics (cORVIS) is an inevitable approach to determining the true dynamic response of the cornea. Such high-order mapping can not only more accurately reproduce the nonlinear oscillation of the cornea under high-speed airflow but also provide a novel physical dimension for understanding the potential role of aqueous humor dynamics in the pathogenesis of glaucoma and corneal ectasia (Safdel and Hassani, 2021; Redaelli et al., 2022; Chen et al., 2024).

Meanwhile, the inherent contradiction between the computational cost of finite element analysis and the clinical demand for real-time feedback constitutes another formidable bottleneck. For high-precision patient-specific models, especially simulations involving anisotropic constitutive laws and complex surgical procedures, a single computation often takes hours or even days, which is clearly impractical in surgical navigation or outpatient screening scenarios requiring second-level auxiliary decision-making.

To address the computational bottleneck of traditional FEA, the integration of artificial intelligence (AI) has shifted from theoretical exploration to practical implementation in corneal biomechanics. Recent studies demonstrate that AI can move beyond simple “black-box” predictions to assist in critical modeling stages, including boundary condition estimation, geometry reconstruction, and inverse parameter calibration.

Regarding loading and boundary conditions, Desouky et al. successfully replaced computationally expensive fluid-structure interaction (FSI) simulations with a Gradient Boosting Regressor (GBR) to predict patient-specific air-puff pressure distributions. Their model achieved a high correlation (*R* ^2^ > 0.98) with numerical CFD results, enabling the rapid generation of accurate loading profiles for personalized FE models. In terms of geometry and microstructure, Gururani et al. utilized birefringence analysis and automated identification algorithms to determine subject-specific fibrillar disposition, providing a high-fidelity structural basis for FE meshes, which accounts for individual variations in collagen orientation (Desouky et al., 2023; Gururani et al., 2023).

The most significant progress lies in material parameter calibration. Traditional inverse FEA requires hundreds of iterations. However, Redaelli et al. proposed a Proper Orthogonal Decomposition-Neural Network (POD-NN) methodology that identifies HGO constitutive parameters (e.g., *C*_10_, *k*_1_) from Corvis ST deformation data in less than one second, with a mean error of only 3.5%. Similarly, Muench et al. demonstrated a “mechanical morphing” approach that identifies degenerated material property ranges in keratoconic eyes almost in real-time (minutes), providing clinical utility for disease staging. This transition toward surrogate modeling, such as the adaptive sparse Polynomial Chaos Expansion (PCE) used by Yaghoubi et al. to estimate IOP and dynamic corneal responses, allows for high-fidelity “offline training” and “online inference.” Such efficiency is essential for integrating biomechanical logic into wearable clinical monitors, such as the smart glasses for IOP tracking proposed by Zolfaghari et al. (Muench et al., 2022; Yaghoubi et al., 2022; Zolfaghari et al., 2023; Redaelli et al., 2025).

Figure 7 shows a schematic diagram of an AI-accelerated corneal digital twin architecture. On the left, inputs include patient-specific measurements such as Corvis-ST deformation curves, the air jet device for air-puff tonometry, and corneal tomographic/polarimetric imaging. The blue area in the middle represents the AI acceleration modules: gradient boosting regression (GBR) for fast prediction of air-jet pressure distribution (replacing traditional CFD); birefringence imaging to obtain individual collagen fibre orientation; the POD-NN method for second-scale inversion of HGO material parameters from Corvis-ST data; and a mechanical deformation method to estimate keratoconus degradation parameters within minutes. In addition, an adaptive sparse PCE surrogate model enables offline training and online inference for rapid IOP and dynamic response prediction. All AI modules feed geometry, loading and material information into a fast FE simulation, generating personalised corneal deformation and IOP predictions in real time, supporting clinical applications such as smart glasses for real-time IOP monitoring. The above results demonstrate the feasibility of using AI to replace computationally intensive steps in traditional FE, providing an acceleration solution for clinical-grade digital twins (Ariza-Gracia et al., 2016; Muench et al., 2022; Desouky et al., 2023; Redaelli et al., 2025; Zhang et al., 2026).

**FIGURE 7:**
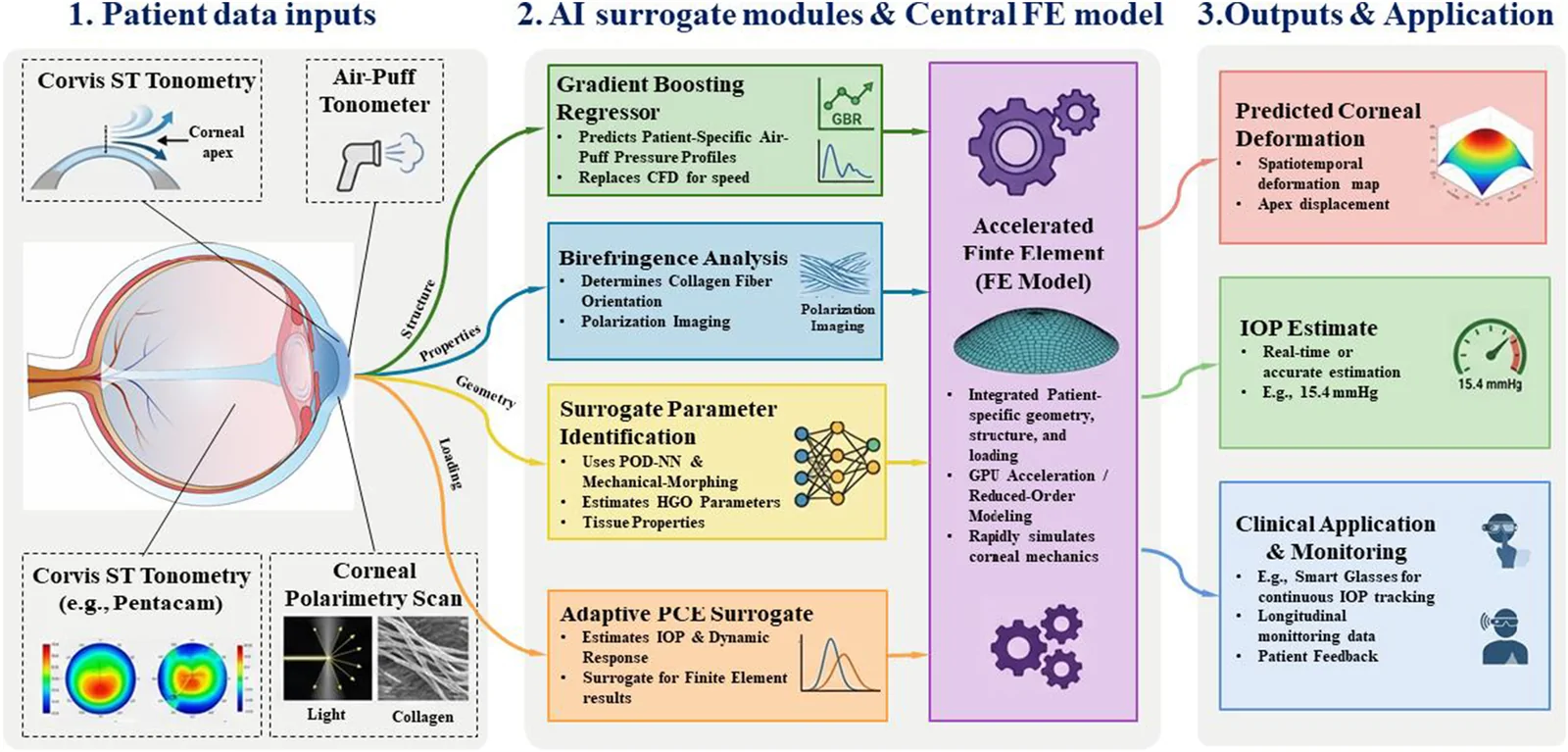
Workflow for the digital twin of corneal biomechanics. Original figure created by the authors based on the scientific information reported in Ariza-Gracia et al. (2016), Muench et al. (2022), Desouky et al. (2023), Redaelli et al. (2025), and Zhang et al. (2026).

The transition from retrospective verification to preoperative planning integrates biomechanical analysis into clinical workflows, facilitating more precise surgical design. While safeguarding the safety of refractive surgery, it will also open up an alternative approach for the precise management of human visual health (Ji et al., 2023; Chen et al., 2024; Kory et al., 2024; Redaelli et al., 2024).

## 5 Conclusion

In conclusion, finite element modeling has matured into a robust, clinically relevant platform that shifts our understanding of corneal biomechanics from empirical observation to precise, predictive simulation. By progressing from simplified geometries and basic material laws to fully personalized, multi-physics digital twins, these models now deliver actionable biomechanical data that directly support individualized refractive surgery planning, refined cross-linking protocols, and optimized transplantation outcomes. As computational efficiency improves and integration with real-time clinical imaging becomes routine, corneal finite element analysis is poised to move from specialized research settings into everyday ophthalmic practice, ultimately enhancing procedural predictability, reducing complications, and providing safer, vision-preserving care for patients worldwide.
